# Antineoplastic effects of rosiglitazone and PPAR*γ* transactivation in neuroblastoma cells

**DOI:** 10.1038/sj.bjc.6603344

**Published:** 2006-09-12

**Authors:** I Cellai, S Benvenuti, P Luciani, A Galli, E Ceni, L Simi, S Baglioni, M Muratori, B Ottanelli, M Serio, C J Thiele, A Peri

**Affiliations:** 1 Endocrine Unit, Department of Clinical Physiopathology, Center for Research, Transfer and High Education on Chronic, Inflammatory, Degenerative and Neoplastic Disorders (DENOThe), University of Florence, Florence, Italy; 2 Gastroenterology Unit, Department of Clinical Physiopathology, Center for Research, Transfer and High Education on Chronic, Inflammatory, Degenerative and Neoplastic Disorders (DENOThe), University of Florence, Florence, Italy; 3 Clinical Biochemistry Unit, Department of Clinical Physiopathology, Center for Research, Transfer and High Education on Chronic, Inflammatory, Degenerative and Neoplastic Disorders (DENOThe), University of Florence, Florence, Italy; 4 Andrology Unit, Department of Clinical Physiopathology, Center for Research, Transfer and High Education on Chronic, Inflammatory, Degenerative and Neoplastic Disorders (DENOThe), University of Florence, Florence, Italy; 5 Pediatric Oncology Branch, Center for Cancer Research, National Cancer Institute, National Institutes of Health, Bethesda, MD, USA

**Keywords:** PPAR*γ*, rosiglitazone, neuroblastoma

## Abstract

Neuroblastoma (NB) is the most common extracranial solid tumour in infants. Unfortunately, most children present with advanced disease and have a poor prognosis. In the present study, we evaluated the role of the peroxisome proliferator-activated receptor *γ* (PPAR*γ*) agonist rosiglitazone (RGZ) in two NB cell lines (SK-N-AS and SH-SY5Y), which express PPAR*γ*. Rosiglitazone decreased cell proliferation and viability to a greater extent in SK-N-AS than in SH-SY5Y. Furthermore, 20 *μ*M RGZ significantly inhibited cell adhesion, invasiveness and apoptosis in SK-N-AS, but not in SH-SY5Y. Because of the different response of SK-N-AS and SH-SY5Y cells to RGZ, the function of PPAR*γ* as a transcriptional activator was assessed. Noticeably, transient transcription experiments with a PPAR*γ* responsive element showed that RGZ induced a three-fold increase of the reporter activity in SK-N-AS, whereas no effect was observed in SH-SY5Y. The different PPAR*γ* activity may be likely due to the markedly lower amount of phopshorylated (i.e. inactive) protein observed in SK-N-AS. To our knowledge, this is the first demonstration that the differential response of NB cells to RGZ may be related to differences in PPAR*γ* transactivation. This finding indicates that PPAR*γ* activity may be useful to select those patients, for whom PPAR*γ* agonists may have a beneficial therapeutic effect.

Thiazolidinediones (TZDs) are a class of molecules, which activate the nuclear receptor peroxisome proliferator-activated receptor *γ* (PPAR*γ*) (Desvergne and Wahli, 1999) and promote association with the 9-*cis* retinoic X receptor (RXR) to form functional heterodimers that recognise its cognate DNA response element within target genes (Jude-Aubry *et al*, 1997; Reginato *et al*, 1998). In addition to their well-known effects on glucose homeostasis (Yki-Järvinen, 2004), TZDs have been shown to have anti-inflammatory (Jiang *et al*, 1998, Marx *et al*, 2000) and antineoplastic effects (Koeffler, 2003, Grommes *et al*, 2004). The latter effect is in agreement with the demonstration that PPAR*γ*/RXR signalling exerts an important role in inhibiting cell proliferation and/or in inducing apoptosis (Grommes *et al*, 2004). In addition, loss-of-function mutations of the PPAR*γ* gene have been found in human colon and thyroid cancer (Sarraf *et al*, 1999; Kroll *et al*, 2000). Therefore, PPAR*γ* has been regarded as a target for anticancer therapy and clinical trials with TZDs for the treatment of human malignancies involving different organs and tissues, such as the prostate (Hisatake *et al*, 2000; Mueller *et al*, 2000), the colon (Kulke *et al*, 2002), the breast (Burstein *et al*, 2003) and the adipose tissue (Demetri *et al*, 1999; Debrock *et al*, 2003) have been initiated in recent years. Peroxisome proliferator-activated receptor *γ* is expressed in tumours of the nervous system, such as astrocytomas, glioblastomas and neuroblastomas (Han *et al*, 2001; Nwako and Robbins, 2001; Strakova *et al*, 2004). Neuroblastoma (NB) is the most common extracranial solid tumour in children and it has a heterogeneous clinical presentation and course (Shimada and Joshi, 1997). Unfortunately, most children with NB present with advanced disease. More than 60% of patients with high-risk features will have a poor prognosis despite intensive therapy. Thus, research efforts to understand the biologic basis of NB and to identify new and more effective therapies are essential to improve the outcome for these children. Retinoids have been shown for instance to interfere with cell growth and to induce apoptosis in NB cells (Melino *et al*, 1997, Voigt and Zintl, 2003) and preliminary clinical trials with retinoids in NB resulted in improved event-free survival in high-risk patients, with limited toxic effects (Garaventa *et al*, 2003; Reynolds *et al*, 2003).

The aim of our study was to evaluate the role of the PPAR*γ* agonist rosiglitazone (RGZ) on cell growth, adhesion, invasiveness and apoptosis in two different NB cell lines (SK-N-AS and SH-SY5Y), which express PPAR*γ* (Servidei *et al*, 2004, Valentiner *et al*, 2005). Furthermore, transient transfection experiments with a peroxisome proliferator response element-luciferase reporter plasmid were performed, in order to determine whether cell response to RGZ was related to the level of PPAR*γ* transcriptional activity. Finally, gene sequencing of the PPAR*γ* gene was performed and the amount of expression of PPAR*γ* (total and phosphorylated) was evaluated and correlated to its transcriptional activity in the two NB cell lines.

## MATERIALS AND METHODS

### Materials

Human NB cell lines SH-SY5Y and SK-N-AS (American Type Culture Collection, Manassas, VA, USA) were made available by the laboratory of Dr CJ Thiele. All the reagents for cell cultures were from Sigma Chemical Co. (St Louis, MO, USA). Tissue plastic-ware was from Bibby Sterilin (Staffordshire, UK). Rosiglitazone was purchased from Alexis (San Diego, CA, USA).

### Cell proliferation

DNA synthesis in NB cells was evaluated by ^3^[H]-thymidine incorporation assay. The cells were seeded in 24-well plates and treated with RGZ for 24 and 48 h. During the last 4 h, the cells were pulsed with 1 *μ*Ci well^−1^ ^3^[H]-thymidine (Amersham Biosciences, Little Chalfont, Buckinghamshire, UK). Finally, the cells were washed with PBS and fixed by cold trichloroacetic acid (5% in distilled water). A 600 *μ*l well^−1^ portion of extraction buffer (NaOH 0.25 N/SDS 0.1%) was added for 30 min at 37°C for DNA extraction. Successively, after pH neutralisation with 6 N HCl, the samples were analysed by a *β*-counter (Betamatic V, Kontron Instruments Ltd, Bletchley, UK). The PPAR*γ* antagonist 2,2-bis[4-(glycidyloxy)phenyl]propane (BADGE, Sigma, Milan, Italy) was used at the concentration of 20 *μ*M and it was added to the cultured cells 30 min before RGZ exposure.

For cell proliferation assay, the cells were seeded in six-well plates. After 24 h, the cells were washed with PBS and different doses of RGZ were added in RPMI medium 1% FBS for 24 or 48 h. Then, the cells were detached (trypsin 0.25%/EDTA) and the cell number was determined by a haemocytometer.

### Cell viability

Cell viability following exposure to different RGZ doses (0.1–20 *μ*M) was determined by MTS assay (Promega Corporation, Madison, WI, USA), as described previously (Benvenuti *et al*, 2005). Briefly, the cells were seeded in 96-well plates and exposed to RGZ for 48 h. The assay was then performed according to the manufacturer's instructions. The samples were analysed by an ELISA plate reader (Seac-Radim, Moncalieri, Italy) at 490 nm wavelength. The PPAR*γ* antagonist BADGE (Sigma) was used at the concentration of 20 *μ*M and it was added to the cultured cells 30 min before RGZ exposure.

### Cell adhesion assay

The cells were plated in 96-well plates and different doses of RGZ (0.1–20 *μ*M) were added for 24 and 48 h. After washing with PBS, 100 *μ*l of Bengal Rose stain (0.25% in PBS) was added to each well for 5 min at room temperature (Gamble and Vadas, 1988). After aspiration of the stain and washing with PBS, the stain was released by adding 200 *μ*l well^−1^ of an ethanol/PBS (1 : 1) solution for 30 min. The samples were analysed by an ELISA plate reader (Seac-Radim) at 570 nm wavelength.

### Invasion assay

Cell invasion was evaluated using the Chemicon Cell Invasion Assay (Temecula, CA, USA). The assay is performed in a 24-well tissue culture plate with 12 cell culture inserts and each Invasion Chamber insert contains an 8 *μ*m pore size membrane over which a thin layer of extracellular matrix is dried. The cells were suspended in serum-free medium and RGZ (20 *μ*M) was added. Successively, after rehydration of the insert, the cells were plated in each Invasion Chamber insert. After 24 h, the noninvading cells on the upper surface of the filter were removed, whereas the invasive cells were stained with the Cell Stain Solution provided with the kit, eluted with 150 *μ*l insert^−1^ of 10% acetic acid, transferred onto a microplate and analysed by an ELISA plate reader (Seac-Radim) at 540 nm wavelength.

### Quantitation of matrix metalloproteinase-9 (MMP-9) and tissue inhibitor of matrix metalloproteinase-1 (TIMP-1) mRNA

The measurement of MMP-9 and TIMP-1 mRNA was performed by using a multiplex quantitative real-time RT–PCR method, based on TaqMan technology, as described previously (Cioppi *et al*, 2004). Standard curves for MMP-9 and TIMP-1 mRNAs consisted of serial 1 : 10 dilutions from 2.5 × 10^7^ to 2.5 × 10^2^ fg total RNA from the fibrosarcoma cell line HT1080 (American Type Culture Collection).

### ELISA assay for MMP-9

Matrix metalloproteinase-9 was measured in conditioned medium using an ELISA-based commercial kit (Pharmacia, Uppsala, Sweden), following the manufacturer's instructions. Matrix metalloproteinase-9 can be measured in a range of 1–32 ng ml^−1^, and the sensitivity of the assay is 0.6 ng ml^−1^.

### Immunocytochemistry and flow cytometry analysis for cleaved caspase 3

The amount of caspase 3 immunoreactive cells was determined as described previously (Benvenuti *et al*, 2005). Briefly, the cells were seeded in chamber slides and exposed to RGZ (20 *μ*M). After 48 h, the cells were fixed in paraformaldehyde, then incubated with a rabbit polyclonal antibody against human cleaved caspase 3 (Asp 175) (Cell Signalling Technology Inc., Beverly, MA, USA) and subsequently with a biotinylated secondary antibody. The reaction product was visualised by ABC peroxidase-based detection protocol and AEC kit (Vectastain ABC kit, Vector Laboratories Inc., Burlingame, CA, USA). Finally, the cells were counterstained with haematoxylin and apoptotic cells/field were counted in 10 fields (× 40).

Moreover, cleaved caspase 3 was evaluated by flow cytometry, which was performed as described previously (Carloni *et al*, 1998). Briefly, the cells were seeded onto 100 mm Petri dishes at the density of 3 × 10^6^ cells and incubated for 24 h in complete medium. Then, RGZ (20 *μ*M) was added and the cells were incubated at 37°C in humidified atmosphere (5% CO_2_/95% air). After 48 h, the cells were detached (trypsin 0.25%/EDTA) and fixed with 3% paraformaldehyde in distilled H_2_O for 10 min at 37°C. After permeabilisation in ice-cold 90% methanol, the cells were incubated with a rabbit polyclonal antibody against cleaved caspase 3 (Asp 175) (Cell Signalling Technology) or with a nonspecific IgG as control at the appropriate dilution (1 : 25) in Incubation Buffer (BSA 0.5% in PBS 1 ×). Subsequently, the cells were washed with Incubation Buffer and incubated with a FITC-conjugated goat anti-rabbit secondary antibody (1 : 100) (Southern Biotechnology Associates Inc., Birmingham, AL, USA) for 30 min. FITC green fluorescence was detected at 515–555 nm using a FL-1 detector of a FACScan flow cytometer (Becton Dickinson, Mountain View, CA, USA) equipped with a 15 mW argon-ion laser for excitation. Debris were gated out by establishing a region around the population of interest on the Forward Scatter *vs* Side Scatter dot plot. For each sample, 10 000 events in the region of interest were recorded at a flow rate of 200–300 cells/s^−1^. Data were processed with analysis software LYSYS II (Becton Dickinson).

### Transient transfection of NB cells

In order to determine the activity of PPAR*γ* in NB cells, the cells were transfected at the density of 4 × 10^6^ cells (SK-N-AS) and 2 × 10^6^ cells (SH-SY5Y) in 100-mm dishes with 2 *μ*g peroxisome proliferator response element (adipocyte response element)-7_3_-tk-luciferase reporter plasmid (containing three copies of the peroxisome proliferator response element from the adipocyte lipid binding protein gene ligated to a herpes simplex thymidine kinase promoter upstream from a luciferase gene), 0.15 *μ*g of human PPAR*γ* expression plasmid, and 1 *μ*g pSV_2_CAT (a vector containing Simian virus 40 early promoter and enhancer sequences that drives a chimeric chloramphenicol acetyl transferase gene) as an internal control by calcium phosphate precipitation, as described previously (Ferruzzi *et al*, 2005). Twenty-four hours after transfection, the cells were treated with RGZ. Twenty-four hours later, the cells were harvested, washed twice with PBS and lysed. Fifty microlitres of cell extract was incubated with luciferase assay reagent based on the original protocol by de Wet *et al* (1987). The number of relative light units with a 3-s delay and a 30-s incubation was measured using a Sirius1 luminometer (Berthold Detection System, Pforzheim, Germany). Chloramphenicol acetyl transferase activity was measured as described previously (Crabb and Dixon, 1987). The conversion of chloramphenicol to its acetylated products was quantified on a *β*-scanner (Ambis System, San Diego, CA, USA).

### Sequence analysis of the PPAR*γ* gene

Genomic DNA was extracted from cell cultures using the automated DNA extraction system Biorobot EZ1 Workstation (Qiagen, Crawley, W Sussex, UK). The entire coding region of the PPAR*γ* gene and the exon–intron boundaries were amplified by PCR, using primers sequences and PCR conditions as described previously (Costa-Guda *et al*, 2005). Amplicons were purified using the QIAquick purification kit (Qiagen) and direct sequencing was carried out using the Big Dye Terminator v1.1 Cycle Sequencing kit (Applied Biosystems, Warrington, Cheshire, UK), according to the manufacturer's instructions. Sequencing reactions were purified using the DyeEx 2.0 Spin kit (Qiagen) and samples were run on an ABI 310 capillary sequencer (Applied Biosystems).

### Western blot analysis of PPAR*γ* expression

The NB cells were washed and resuspended in lysis buffer. In addition, the human adenocarcinoma cell line PANC-1 and HPAC (American Type Culture Collection) were used as the positive and negative control for PPAR*γ* expression, respectively (Galli *et al*, 2004). After protein measurement (Coomassie kit, Bio-Rad Laboratories, Hercules, CA, USA), 30 *μ*g of protein was diluted in 2 × Laemmli's reducing sample buffer, incubated at 95°C for 5 min and loaded onto a 10% polyacrylamide-bisacrylamide gel. After SDS–PAGE, proteins were electroblotted into nitrocellulose (Sigma Chemical Co.). Equivalent protein loading was verified by staining parallel gels with Coomassie R. After blocking in 5% skimmed milk for 2 h, nitrocellulose membranes were washed and then immunostained with a rabbit anti-human PPAR*γ* antibody (1 : 1000) (Santa Cruz Biotechnology, Santa Cruz, CA, USA) followed by a secondary anti-mouse IgG antibody (1 : 2000) (New England Biolabs, Beverly, MA, USA). The antibody-reacted proteins were revealed by LumiGLO chemiluminescent reagent and peroxide (New England Biolabs).

### PPAR*γ* phosphorylation assay

Protein extraction was performed by boiling the cell pellet in 10% glycerol, 2% SDS and 50 mM Tris-HCl (pH 7.5) for 10 min. The lysate was diluted 1 : 10 with RIPA-like buffer containing 50 mM Tris-HCl (pH 7.5), 1% Triton X-100, 0.5% sodium deoxycholate, 150 mM NaCl, 5 mM EDTA, 25 mM *β*-glycerophosphate, 1 mM Na_3_VO_4_, 100 *μ*M PMSF, supplemented with Complete Protease Inhibitor Cocktail (Roche Applied Science, Milan, Italy). SDS was then added to achieve a final concentration of 1% and immunoprecipitation was performed with a rabbit anti-human PPAR*γ* antibody (1 : 100) (Santa Cruz Biotechnology) at 4°C, for 3 h. Immunocomplexes were recovered by incubation with Protein-A Sepharose (Sigma) for additional 16 h at 4°C. The immunocomplexes were then dissociated by boiling for 5 min in Laemmli's buffer, the beads were collected by centrifugation and SDS–PAGE was performed with the supernatant. The protein were electroblotted to nitrocellulose transfer membrane Protean (Whatman, VWR International, Milan, Italy) and were detected by incubating the filter with the anti-PPAR-*γ* antibody or with an anti-phosphoserine mouse monoclonal antibody (1 : 400) (clone PSR-45, Sigma) followed by a secondary anti-rabbit IgG (1 : 2000) or anti-mouse IgG (1 : 2000) antibody, respectively (Amersham Biosciences). Detection of the protein bands was performed using the Amersham ECL plus Kit (Amersham Biosciences).

### Statistical analysis

All the experiments were carried out in triplicate or esaplicate and were repeated at least three times. Data were expressed as mean±s.e. Statistical differences were analysed using one-way analysis of variance. Significance was adjusted for multiple comparisons of means using Bonferroni's approximation.

## RESULTS

### Effects of RGZ treatment on cell proliferation

**Figure 1:**
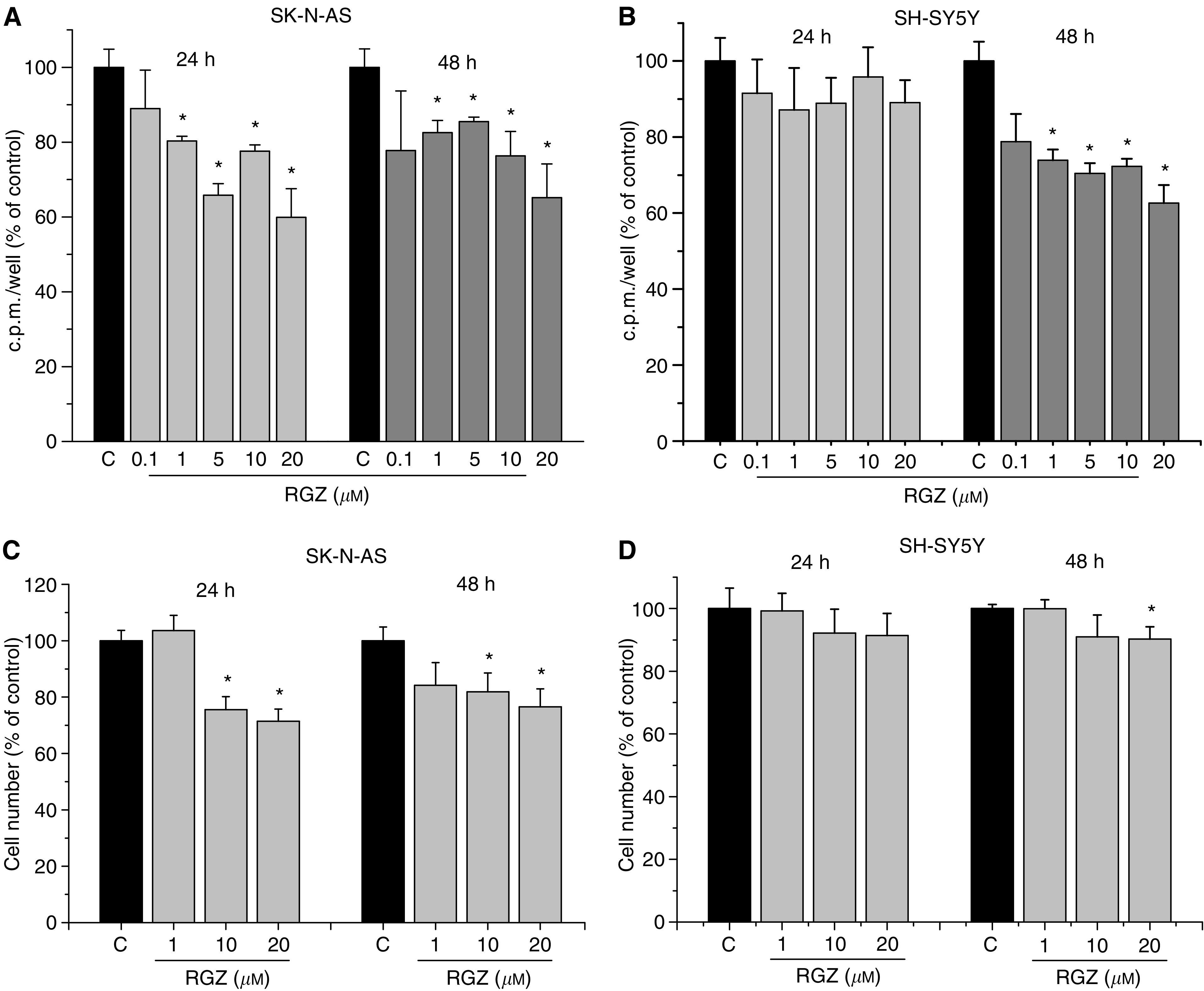
(**A** and **B**) Effect of a 24- or a 48-h treatment with RGZ on ^3^[H]thymidine incorporation in SK-N-AS (**A**) and SH-SY5Y (**B**) cells. ^*^P<0.05 *vs* control untreated cells (C). (**C** and **D**) Effect of a 24- or a 48-h treatment with RGZ on SK-N-AS (**C**) and SH-SY5Y (**D**) cell proliferation. ^*^P<0.05 *vs* control untreated cells (C).

The effects of RGZ on ^3^[H]-thymidine incorporation in the two NB cell lines SK-N-AS and SH-SY5Y are reported in Figure 1. In SK-N-AS (Figure 1A), a significant reduction of DNA synthesis was determined by exposure to RGZ (1–20 *μ*M) for 24 and 48 h, whereas in SH-SY5Y (Figure 1B), this effect was observed only after a 48-h exposure.

With regard to cell counts, a significant antiproliferative effect was observed following a 24- or 48-h treatment with RGZ (10 and 20 *μ*M) in SK-N-AS (Figure 1C). In SH-SY5Y, a significant reduction of the cell number was observed only after a 48-h treatment with 20 *μ*M RGZ (Figure 1D), although to a lower extent than in SK-N-AS. Only a higher concentration of RGZ (80 *μ*M) was able to determine in SH-SY5Y a reduction of the cell number (54.4±1.3%, mean±s.e. *vs* untreated cells, considered as 100%) similar to the decrease determined by the same dose of RZG in SK-N-AS (57.9±1.4%) (data not shown).

### Effects of RGZ on cell viability and adhesion

**Figure 2:**
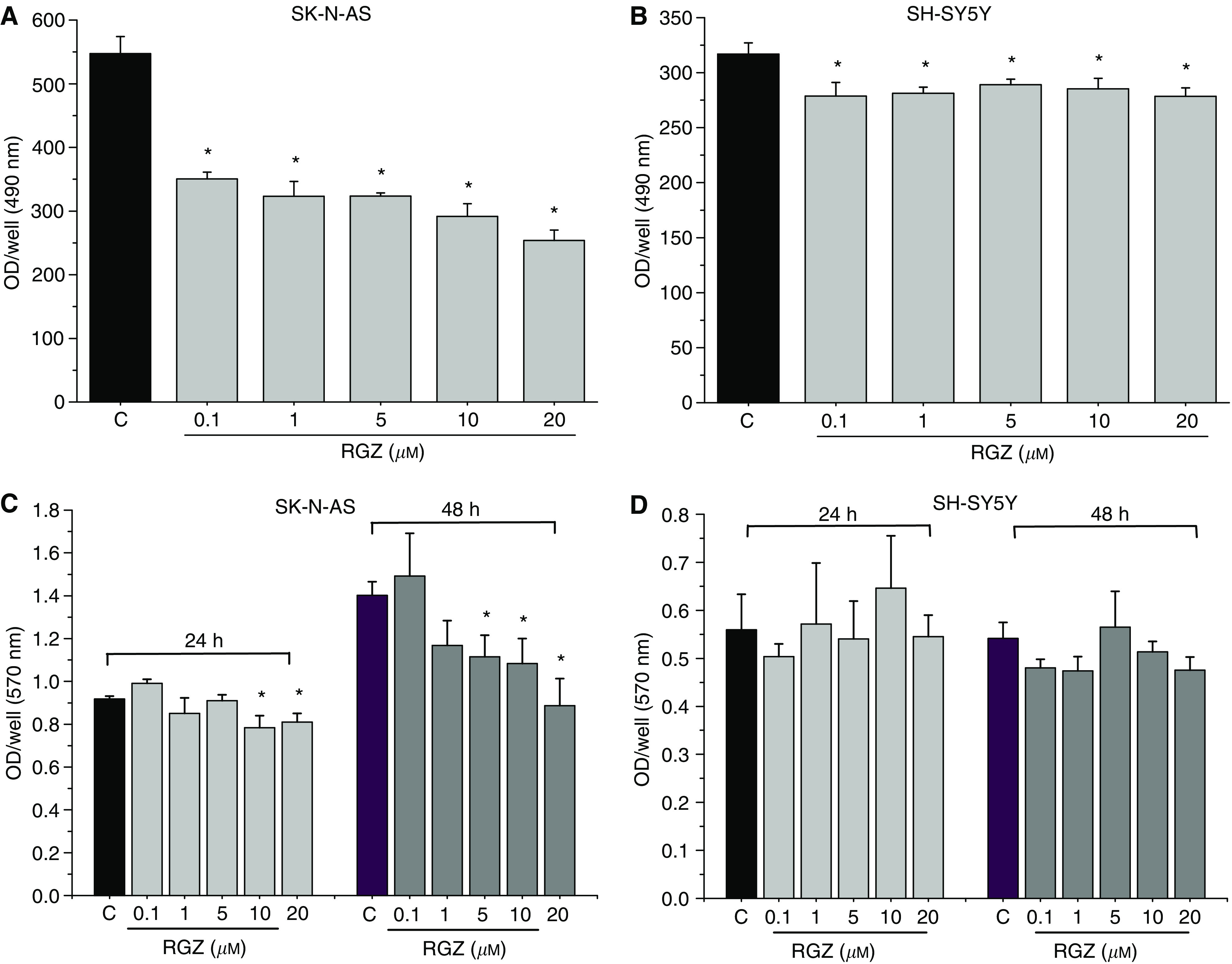
(**A** and **B**) Role of RGZ on SK-N-AS (**A**) and SH-SY5Y (**B**) cell viability, as assessed by MTS assay (see Materials and Methods). ^*^P<0.05 *vs* control untreated cells (C). (**C** and **D**) Effect of different concentrations and time of exposure to RGZ (24 or 48 h) on SK-N-AS (**C**) and SH-SY5Y (**D**) cell adhesion, as assessed by Bengal Rose assay (see Materials and Methods). ^*^P<0.05 *vs* control untreated cells (C).

The effect of RGZ on NB cell viability is shown in Figure 2A and B. In SK-N-AS, cell viability was significantly reduced after a 48-h exposure to RGZ (0.1–20 *μ*M) and 20 *μ*M RGZ determined the maximal inhibition (54%) (Figure 2A). In SH-SY5Y cells, RGZ (0.1–20 *μ*M) was also able to reduce cell viability, but the decrease was limited to 13% (20 *μ*M RGZ) (Figure 2B).

Moreover, the effect of RGZ on cell adhesion was determined. Interestingly, in SK-N-AS cells RGZ significantly induced an inhibition of cell adhesion, which was already present after 24 h of incubation at the concentration of 10 and 20 *μ*M (Figure 2C). After a 48-h treatment, a significant inhibitory effect was already present at a lower dose (5 *μ*M). Conversely, in SH-SY5Y cells no inhibitory effect was observed for all the tested RGZ concentrations, neither after a 24-h nor after a 48-h exposure (Figure 2D).

### Effects of the PPAR*γ* antagonist BADGE on cell proliferation and viability

**Figure 3:**
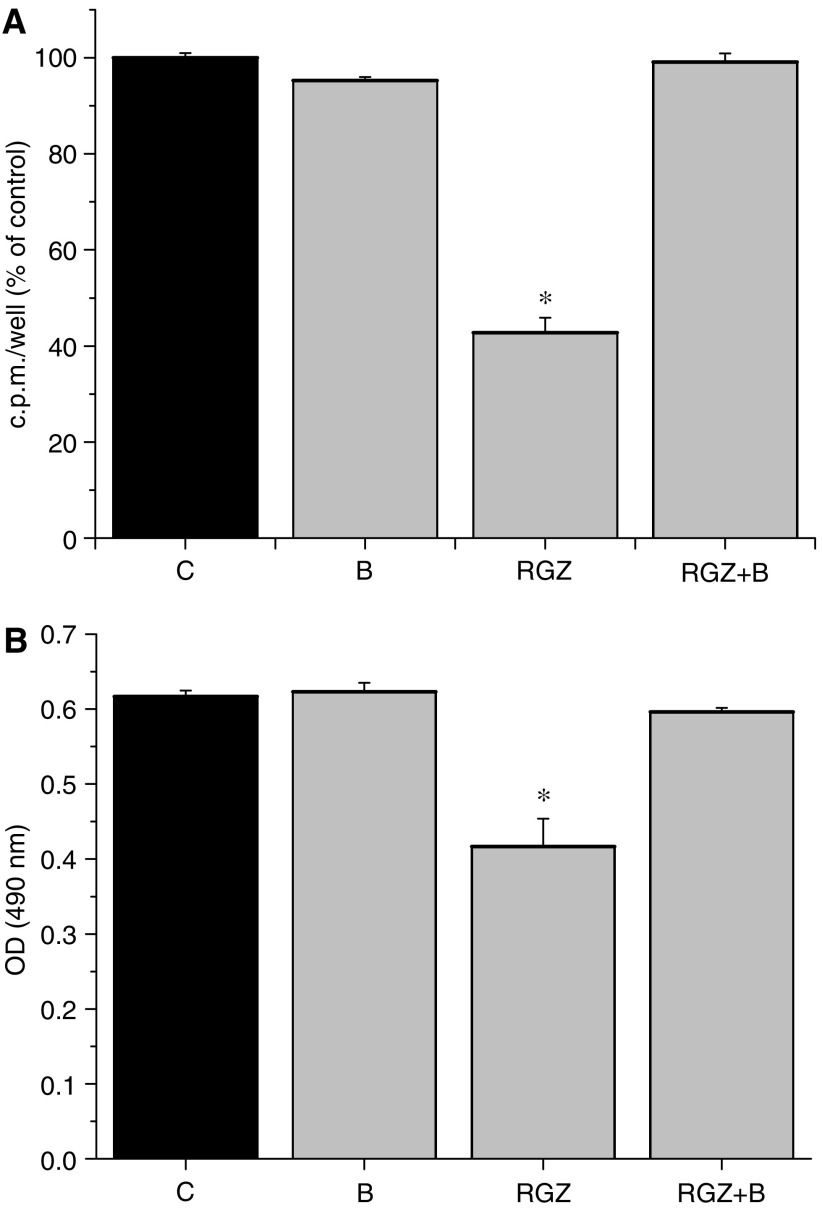
Role of the PPAR*γ* antagonist BADGE (indicated as B) (20 *μ*M) in counteracting the effect on ^3^[H]thymidine incorporation (**A**) and on cell viability (**B**) induced by 20 *μ*M RGZ in SK-N-AS cells. ^*^P<0.05 vs control untreated cells (C).

In order to determine whether the effects of RGZ on cell proliferation and viability were truly mediated by PPAR*γ*, additional experiments were performed in SK-N-AS using the PPAR*γ* antagonist BADGE. The exposure to 20 *μ*M BADGE did not determine any effect both on ^3^[H]-thymidine incorporation and on cell viability, thus indicating that at this concentration this compound had no toxic effect. The same concentration of BADGE was able to revert the inhibitory effect of 20 *μ*M RGZ on ^3^[H]-thymidine incorporation (Figure 3A) as well as on cell viability (Figure 3B).

### Effect of RGZ on cell invasiveness and on the expression of MMP-9/TIMP-1

**Figure 4:**
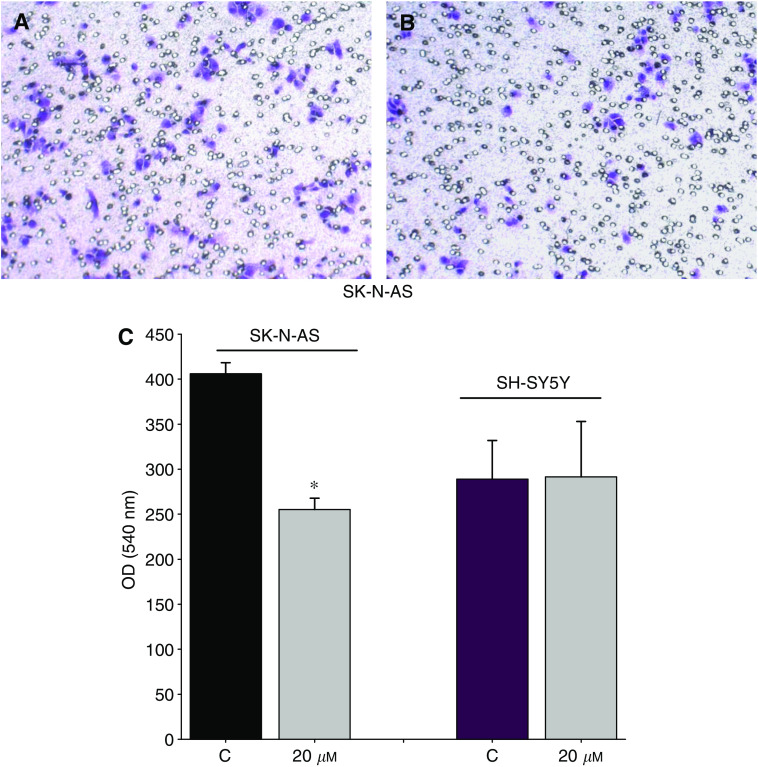
Role of RGZ on SK-N-AS cell invasiveness. (**A**) Untreated control cells. (**B**) Cells treated with 20 *μ*M RGZ for 24 h. The invasive cells on the lower surface of the membrane were stained with crystal violet (see Materials and Methods). (**C**) Spectrophotometric analysis on extracts from stained cells eluted from the membrane invasion chamber. ^*^P<0.05 *vs* control untreated cells (C).

The effect of RGZ (20 *μ*M) on the invasiveness ability of NB cells was determined. When SK-N-AS cells were tested, the number of invasive cells markedly decreased after RGZ exposure. A representative example is shown in Figure 4A and B. At variance with SK-N-AS, no effect was observed in SH-SY5Y (not shown).

These results were quantitatively confirmed by spectrophotometric analysis on extracts from stained cells eluted from the membrane invasion chamber (Figure 4C).

**Figure 5:**
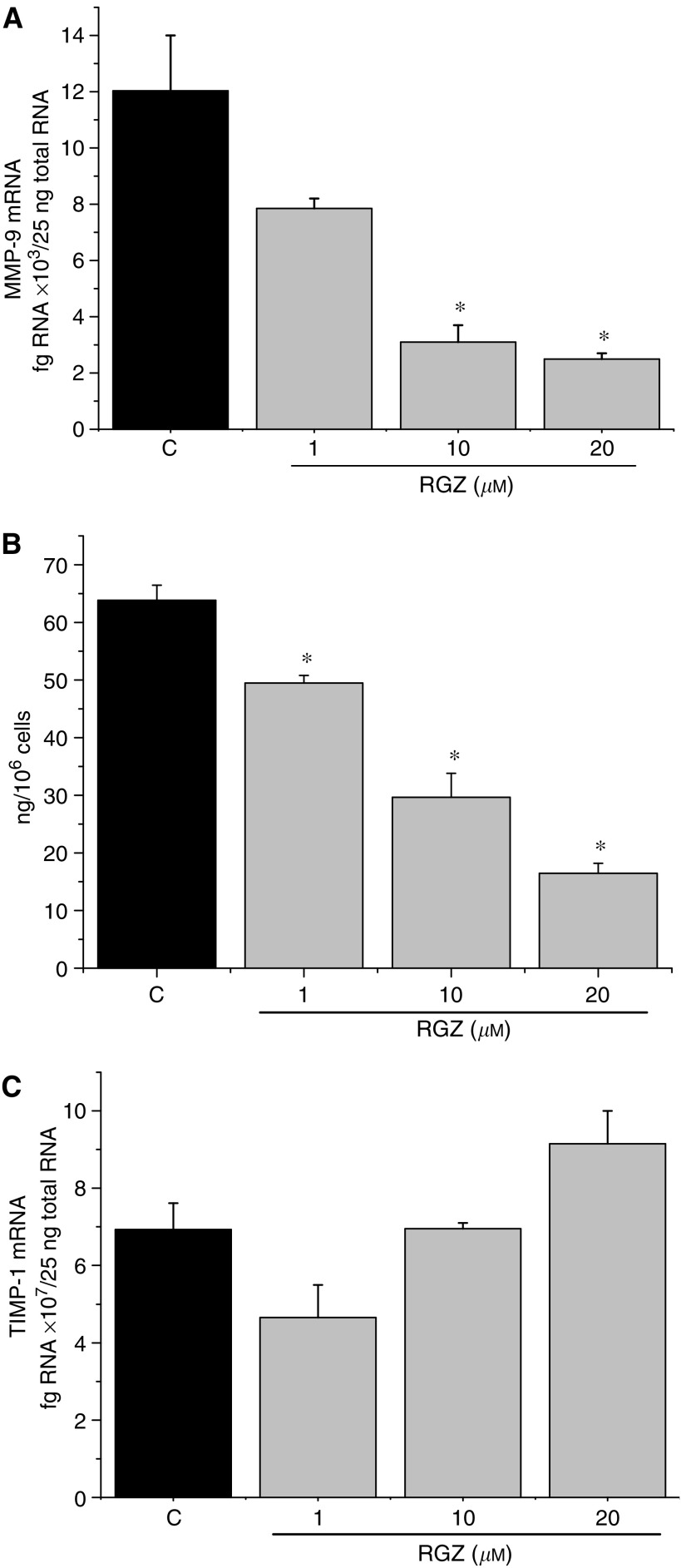
Amount of MMP-9 mRNA (**A**) and protein (**B**) and of TIMP-1 mRNA (**C**) in SK-N-AS cells in the absence or in the presence of different concentrations of RGZ. ^*^P<0.05 *vs* control (indicated as C).

Moreover, in both cell lines the effect of different doses of RGZ (1, 10 and 20 *μ*M) on the expression of MMP-9 and of its specific tissue inhibitor TIMP-1 was investigated. In untreated SK-N-AS cells, the amount of MMP-9 mRNA, as assessed by real-time RT–PCR, was 12.03±1.97 × 10^3^ fg 25 ng total RNA^−1^ (mean±s.e.). Rosiglitazone (10 and 20 *μ*M) significantly decreased the level of MMP-9 transcript (Figure 5A). Accordingly, the amount of protein was significantly reduced by RZG in a dose-dependent manner (Figure 5B). In SH-SY5Y cells, no MMP-9 transcript was detectable (data not shown). As per TIMP-1 expression, the amount of mRNA in untreated cells was 6.93±0.67 × 10^7^ fg 25 ng total RNA^−1^ in SK-N-AS and 6.4±0.2 × 10^6^ fg 25 ng total RNA^−1^ in SH-SY5Y cells. Rosiglitazone did not determine any significant variation, although a trend towards an increase was observed in SK-N-AS (Figure 5C). Altogether, these results suggest that RGZ effectively counteracts cell invasiveness in SK-N-AS, but not in SH-SY5Y cells.

### Effects of RGZ on apoptosis

**Figure 6:**
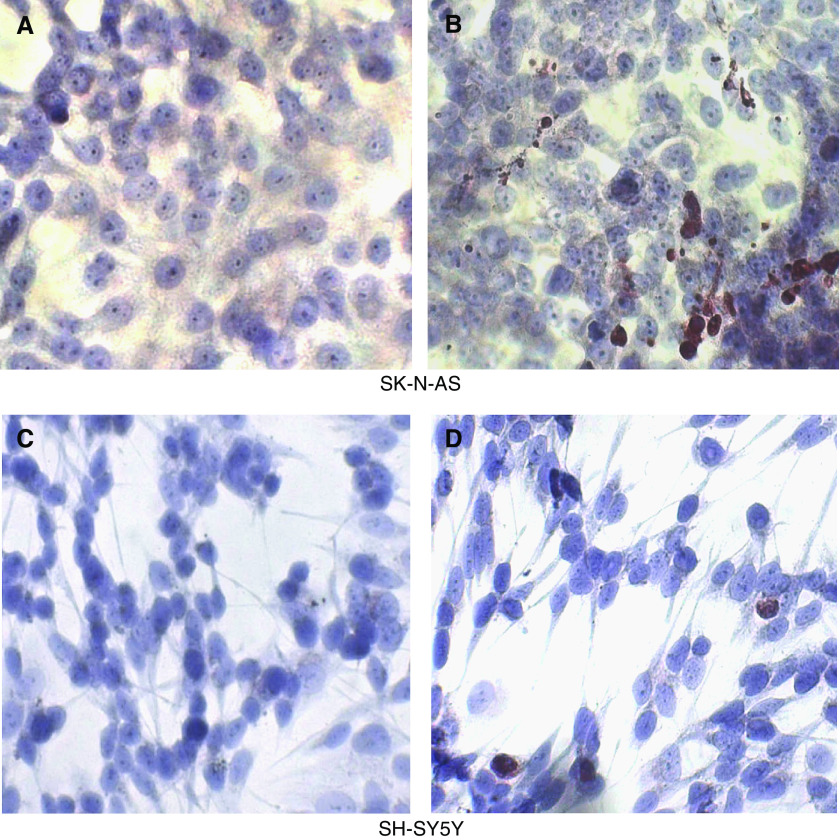
Immunocytochemistry for the detection of cleaved caspase 3-positive cells. (**A** and **C**) Control untreated cells (SK-N-AS and SH-SY5Y, respectively). (**B** and **D**) RGZ-treated cells (SK-N-AS and SH-SY5Y, respectively). Magnification × 40.

In order to determine whether RGZ is also able to induce apoptosis in the two NB cell lines used in this study, the number of cells showing positivity for cleaved caspase 3 was evaluated by immunocytochemistry. In SK-N-AS, the amount of apoptotic cells in untreated samples was 0.44±0.23% (mean±s.e.), whereas RGZ treatment (20 *μ*M) significantly increased the number of caspase 3-positive cells (2.12±0.24%, *P*<0.05). In SH-SY5Y, there was no difference in the amount of cleaved caspase 3-positive cells in untreated *vs* RGZ-treated samples (0.43±0.13 *vs* 0.49±0.11%, respectively, *P*>0.05). Representative examples are shown in Figure 6. Several immunostained SK-N-AS cells were present after exposure to RGZ (Figure 6B), compared to the absence of staining in untreated cells (Figure 6A). In contrast, only a very few SH-SY5Y cells showed positivity for cleaved caspase 3 either before (Figure 6C) or after (Figure 6D) RGZ treatment. The effect of treatment with RGZ (20 *μ*M) on caspase 3 activation in SK-N-AS was confirmed by flow cytometry analysis. After labelling activated caspase 3, treated cells showed a shift to higher values of fluorescence, when compared to control cells (9.73, treated *vs* 8.28, control, median values of fluorescence histograms). Conversely, no effect was observed in SH-SY5Y (8.51 treated, *vs* 8.48, control, median values of fluorescence histogram) (data not shown).

### PPAR*γ* activity in NB cells

**Figure 7:**
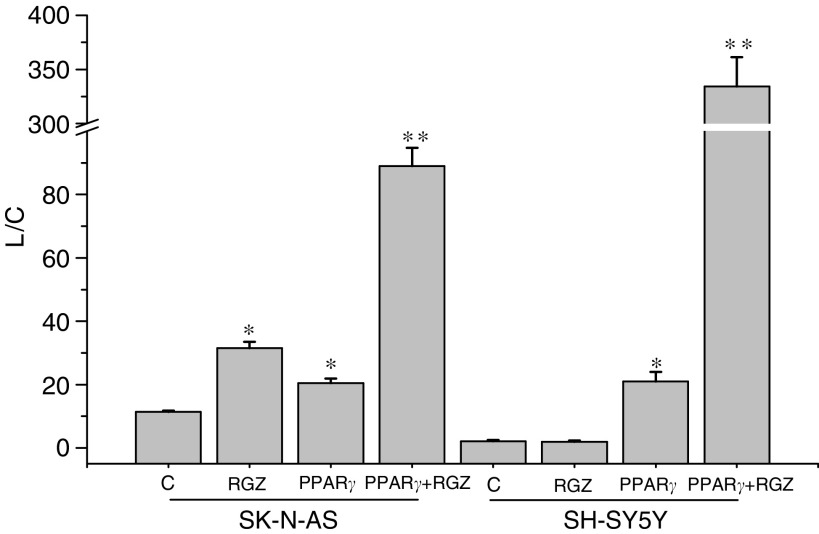
PPAR*γ* transcriptional activity in control untreated NB cells (C), in cells treated with RGZ (20 *μ*M) and in cells transfected with PPAR*γ* in the absence or in the presence of RGZ. L/C: peroxisome proliferator response element-n7_3_-tk-luciferase reporter activity, normalised for CAT activity. ^*^P<0.05 *vs* C. ^**^P<0.05 *vs* PPAR*γ* transfected cells in the absence of RGZ.

Because of the different response to RGZ, PPAR*γ* activity was determined in SK-N-AS and SH-SY5Y cells. Peroxisome proliferator-activated receptor *γ* activity was investigated by transient transfection assay and PPAR*γ* transactivation was monitored by the activity of transfected adipocyte response element-7_3_-tk-luciferase reporter cells. In SK-N-AS, RGZ treatment (20 *μ*M) induced a near three-fold increase of the reporter activity compared to untreated control cells (Figure 7). Conversely, no effect was observed in SH-SY5Y after RGZ exposure. Transfection of NB cell lines with a human PPAR*γ* expression plasmid stimulated the reporter activity about 1.5- and eight-fold over the control in SK-N-AS and SH-SY5Y, respectively. Furthermore, the treatment of PPAR*γ*-transfected cells with RGZ caused an additional five- and 17-fold increase of the reporter activity in the two cell lines, respectively.

### PPAR*γ* gene sequencing and expression in NB cells

**Figure 8:**
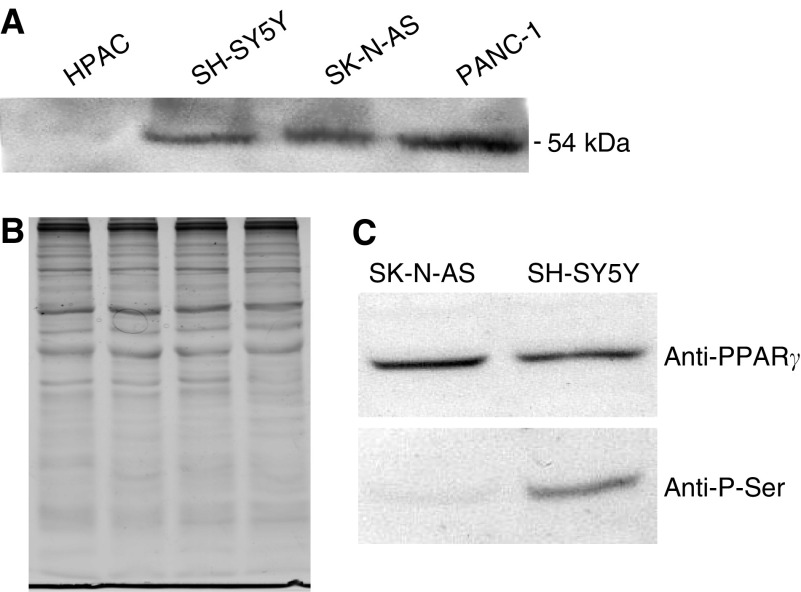
(**A**) Western blot analysis of the amount of PPAR*γ* in SK-N-AS and SH-SY5Y. PANC-1 and HPAC cells (human pancreatic adenocarcinomas) were used as the positive and negative control, respectively. (**B**) Coomassie R-stained gel, showing equal protein loading of the same samples indicated in A. (**C**) Detection of total (anti-PPAR*γ* antibody) and phosphorylated (anti-P-Ser antibody) PPAR*γ*, after PPAR*γ* immunoprecipitation.

In order to determine whether the different function of PPAR*γ* as a transcriptional activator in the two NB cell lines was due to gene mutations, sequence analysis of the entire coding region of the PPAR*γ* gene was performed. However, no mutation was found in either SH-SY5Y or SK-N-AS. Similarly, no evident difference in the amount of expression of PPAR*γ* protein was observed between the two cell lines, as assessed by Western blot analysis (Figure 8A). Equivalent protein loading was verified by staining parallel gels with Coomassie R, as shown in Figure 8B. Because there is evidence that PPAR*γ* phosphorylation reduces the activity of the receptor (Shao and Lazar, 1999), PPAR*γ* immunoprecipitation was performed, followed by SDS–PAGE and Western blot analysis using either an anti-PPAR*γ* or an anti-phosphoserine monoclonal antibody. Interestingly, the amount of phosphorylated PPAR*γ* was found to be markedly lower in SK-N-AS, thus suggesting that the higher PPAR*γ* activity in this cell line compared to SH-SY5Y may be due to a different phosphorylation status of the receptor (Figure 8C).

## DISCUSSION

In the present study, the effect of the PPAR*γ* ligand RGZ on two NB cell lines, which have a stromal (S) (SK-N-AS) and a neuroblast (N) (SH-SY5Y) phenotype (Ciccarone *et al*, 1989; Ross *et al*, 2003; Servidei *et al*, 2004), was addressed. We first demonstrated that cell proliferation was readily inhibited by RGZ in the S-type cell line, whereas this effect was less evident in the N-type cells. Cell viability was significantly reduced by RGZ in both cell lines, although a much stronger maximal inhibition was observed in SK-N-AS. The effects of RGZ on cell proliferation and viability were readily counteracted by the PPAR*γ* antagonist BADGE, thus indicating that PPAR*γ* activation has an important role in eliciting these biological effects. Furthermore, the capability of cultured cells to adhere to a solid substrate in response to RGZ was assessed. In fact, cell adhesion assay is an indicator of cell spreading and adhesion molecules play a critical role in promoting the processes leading to tumour invasion and metastasis (Cavallaro and Christofori, 2004). Again, we found that RGZ readily decreased SK-N-AS cells adhesion starting from a 24-h exposure (10 and 20 *μ*M), whereas it had no effect on SH-SY5Y cells, even after a 48-h treatment. As an additional feature of malignant behaviour, cell invasiveness was determined. Rosiglitazone effectively reduced the passage of the S-type cells, but not of the N-type cells, through the extracellular matrix-coated membranes. Therefore, altogether these results provide evidence that RGZ acts as an effective inhibitor of cell proliferation, adhesion and invasiveness in SK-N-AS, but not in the SH-SY5Y NB cell line.

It has to be mentioned that the inhibitory effect of RGZ on SK-N-AS cell invasiveness might be partially PPAR*γ*-independent. With regard to this point, the activity of MMPs, which promote the invasion of the extracellular matrix by tumoral cells, has been related to the progression of a variety of tumours, including NB (Sugiura *et al*, 1998; Chantrain *et al*, 2004). Accordingly, MMPs have been addressed as a target for cancer therapy (Overall and Kleifeld, 2006). RZG has been shown for instance to reduce the expression/activity of MMPs in a PPAR*γ*-independent manner in adrenal (Ferruzzi *et al*, 2005) and pancreatic tumour cells (Farrow *et al*, 2003; Galli *et al*, 2004) and to increase the expression of TIMP-1 in breast cancer cells (Liu *et al*, 2003). In SK-N-AS, but not in SH-SY5Y, RGZ significantly reduced the amount of MMP-9, whereas the expression of the MMP inhibitor TIMP-1 showed a small but not statistically significant increase at higher levels. The demonstration that the proliferation of embryonic stem cells with a null mutation for PPAR*γ* could be inhibited by glitazones (Palakurthi *et al*, 2001), which were also able to differentiate these cells into macrophages (Chawla *et al*, 2001), further supports the presence of PPAR*γ*-independent mechanisms of agonist action. Furthermore, in our study the role of RGZ in inducing apoptosis in NB cells was addressed. Interestingly, very recently apoptosis has been indicated as a surveillance mechanism against metastases in NB (Stupack *et al*, 2006). In fact, it has been shown that suppression of caspase 8 expression occurs during the development of NB metastases *in vivo*, whereas reconstitution of caspase 8 expression potentiated apoptosis and counteracted the spreading of tumour cells. Peroxisome proliferator-activated receptor *γ* agonists induce apoptosis in different cell types and in most studies it has been shown that caspase 3 plays an important role in mediating apoptotic death (Grommes *et al*, 2004). We found that RGZ (20 *μ*M) increased the amount of cleaved (i.e. activated) caspase 3 only in SK-N-AS cells. However, both immunocytochemistry and flow cytometry experiments did not show a very strong apoptotic effect of RZG, thus suggesting that RGZ does not appear to play a major role in stimulating apoptosis in these NB cells. These results are partially in agreement with a previous report, in which RGZ (up to 50 *μ*M) was not able to induce apoptosis in different NB cell lines, although SK-N-AS cells were not evaluated (Servidei *et al*, 2004).

The different response of SK-N-AS and SH-SY5Y cells to RGZ appeared to be due to the function of PPAR*γ* as a transcriptional activator, which was readily demonstrated in SK-N-AS but not in SH-SY5Y, as assessed by transient transfection with a PPAR*γ* responsive element. This difference might be due to a mutation of the PPAR*γ* gene. No mutation in exons 3 and 5, the biologically important regions for DNA and ligand binding of PPAR*γ*, respectively, has been detected previously in the two NB cell lines, which were used in our study (Servidei *et al*, 2004). A K422Q mutation has been described previously in exon 6 of PPAR*γ* in colon cancer cell lines, but it did not alter ligand-induced transactivation (Gupta *et al*, 2003). Nevertheless, we sequenced the entire coding region of the PPAR*γ* gene in SK-N-AS and in SH-SY5Y and no mutation was found. Only when SH-SY5Y cells were transfected with a human PPAR*γ* expression plasmid, the response to RGZ was effectively elicited, thus confirming that the lack of response was truly due to the very low transactivation potential of the endogenous PPAR*γ*. It has to be mentioned that the phosphorylated status of PPAR*γ* is important in determining its transcriptional activity. In fact, there is evidence that PPAR*γ* phosphorylation reduces the activity of the receptor (Shao and Lazar, 1999). We performed Western blot analysis of total and phosphorylated PPAR*γ* and we found that the amount of the phosphorylated form was markedly lower in SK-N-AS than in SH-SY5Y. This finding indicates that the lower activity of PPAR*γ* in the latter NB cell line may be likely due to a different phosphorylation status of the receptor. In addition, it might be hypothesised that PPAR*γ* overexpression in transfected SH-SY5Y cells might increase the level of unphosphorylated PPAR*γ* beyond a critical threshold, thus eliciting transcriptional activity. Other recent studies have shown variable antineoplastic effects of PPAR*γ* agonists in NB cell lines expressing PPAR*γ* (Han *et al*, 2001, Servidei *et al*, 2004, Valentiner *et al*, 2005), but the activity of PPAR*γ* was addressed only in one study, in which PPAR*γ* was found to be functionally active in different cell lines and particularly in SK-N-AS (Servidei *et al*, 2004). However, the relationship between PPAR*γ* transactivation and the response to TZDs was not addressed. Thus, our study extends the work of other authors, because the different response of NB cells to TZDs was related for the first time to differences in PPAR*γ* transactivation, which, in turn, appear to be due to a different phosphorylation status of the receptor.

Our original results, if confirmed in different cell models, might also open a window on potential prognostic and therapeutic applications, based on PPAR*γ* and PPAR*γ* ligands. The presence in the tumour tissue of a transcriptionally active PPAR*γ* might for instance be useful to select those patients, for whom TZDs might be beneficial. In fact, although some of the effects of TZDs may be PPAR*γ*-independent, as mentioned previously, admittedly most of them are dependent on the presence of a transcriptionally active PPAR*γ*. The low toxicity demonstrated by RGZ as an antidiabetic agent (Yki-Järvinen, 2004) and the fact that the inhibitory effects on cell proliferation, adhesion and invasiveness were present in our study at concentrations similar to those achieved *in vivo* (Cox *et al*, 2000) are additional important points to be raised in favour of the possible use of this drug in NB.
